# Ambulance clinicians’ perceptions, assessment and management of frailty: thematic analysis of focus groups

**DOI:** 10.29045/14784726.2018.12.3.3.23

**Published:** 2018-12-01

**Authors:** Jonathan Green, Kim Kirby, Suzy Hope

**Affiliations:** South Western Ambulance Service NHS Foundation Trust: Orcid ID: 0000-0001-5738-7515; University of the West of England; South Western Ambulance Service NHS Foundation Trust: Orcid ID: 0000-0002-8092-7978; University of Exeter Medical School: Orcid ID: 0000-0001-7343-0149

**Keywords:** emergency medical services, frailty, referral and consultation

## Abstract

**Introduction:**

More than half of all patients attended by the South Western Ambulance Service NHS Foundation Trust are over the age of 65. In 2017, 62% of older patients who were the subject of a frailty assessment were believed to have at least mild frailty (1/5 of all patients). Frailty is an increasingly relevant concept/diagnosis and ambulance services are well positioned to identify frailty and influence the ‘care pathways’ through which patients are directed (thereby influencing health outcomes). Throughout the South Western Ambulance Service NHS Foundation Trust, a mandatory training session regarding frailty was delivered to clinical personnel in 2017 and frailty assessment tools are available on the electronic Patient Clinical Record.

**Aim:**

To explore and gain insight into the current knowledge, practice and attitudes of ambulance clinicians regarding frailty and patients with frailty.

**Methods:**

Two focus groups of ambulance clinicians (n = 8; n = 9) recruited from across the South Western Ambulance Service NHS Foundation Trust were held in October 2017. Focus group discussions were analysed thematically.

**Results:**

Knowledge of conceptual models of frailty, appropriate assessment of patients with frailty and appropriate care pathways varied substantially among focus group participants. Completion of the ‘Rockwood’ Clinical Frailty Scale for relevant patients has become routine. However, conflicting opinions were expressed regarding the context and purpose of this. The Timed-Up-and-Go mobility assessment tool is also on the electronic Patient Clinical Record, but difficulties regarding its completion were expressed.

Patient management strategies ranged from treatment options which the ambulance service can provide, to referrals to primary/community care which can support the management of patients in their homes, and options to refer patients directly to hospital units or specialists with the aim of facilitating appropriate assessment, treatment and discharge. Perceptions of limited availability and geographical variability regarding these referral pathways was a major feature of the discussions, raising questions regarding awareness, capacity, inter-professional relationships and patient choice.

**Conclusion:**

Knowledge, practice and attitudes of ambulance staff, with regard to frailty, varied widely. This reflected the emerging nature of the condition, both academically and clinically, within the ambulance profession and the wider healthcare system.

## Introduction

**Figure 1.**
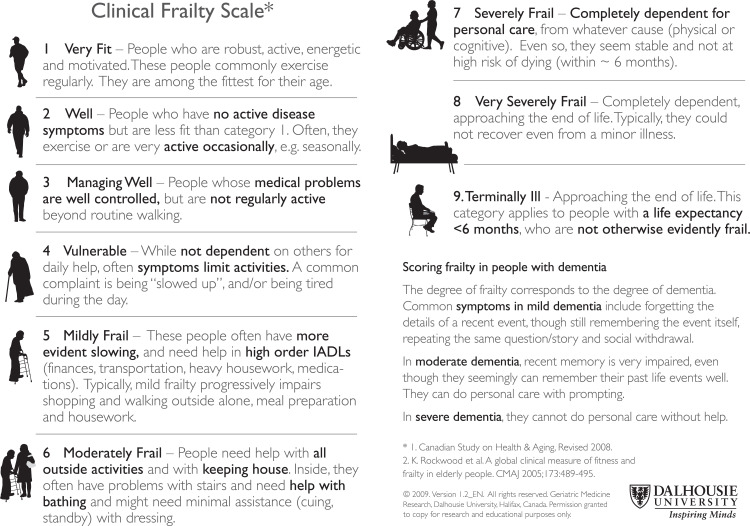
‘Rockwood’ Clinical Frailty Scale (available on SWASFT electronic Patient Clinical Record).

*Source:* Canadian Study of Health and Aging (2008); Rockwood et al. (2005). © 2007–2009. Version 1.2. All rights reserved. Geriatric Medicine. Research, Dalhousie University, Halifax, Canada. Permission granted to copy for research and educational purposes only.

The South Western Ambulance Service NHS Foundation Trust (SWASFT) serves seven English counties from Gloucestershire to Dorset and Cornwall. Fifty-two per cent of emergency calls to SWASFT are for patients over the age of 64 (SWASFT audit, 2018). Approximately two-thirds of these older patients are currently assessed using the ‘Rockwood’ Clinical Frailty Scale (CFS; Figure 1). Where it was applied, 62% were designated a CFS score of 4 or above, suggesting at least mild frailty (NHS England, 2017a). This equates to approximately 135,000 or 1/5 of all SWASFT attendances per year (SWASFT audit, 2017).

Frailty is a long-term condition characterised by the ‘cumulative decline in many physiological systems during a lifetime’ (Clegg, Young, Iliffe, Olde Rikkert, & Rockwood, 2013: 752). Patients with frailty are vulnerable to major deterioration in their physical and mental health as a result of apparently minor events, such as infection, new medication, falls, constipation or urine retention (British Geriatric Society, 2014). Increased levels of frailty are associated with increased mortality, institutionalisation, occurrence of ‘geriatric syndromes’ (e.g. Ensrud et al., 2007; Hubbard et al., 2017; McRae et al., 2016) and post-operative complications (e.g. Jung et al., 2015).

**Figure 2.**
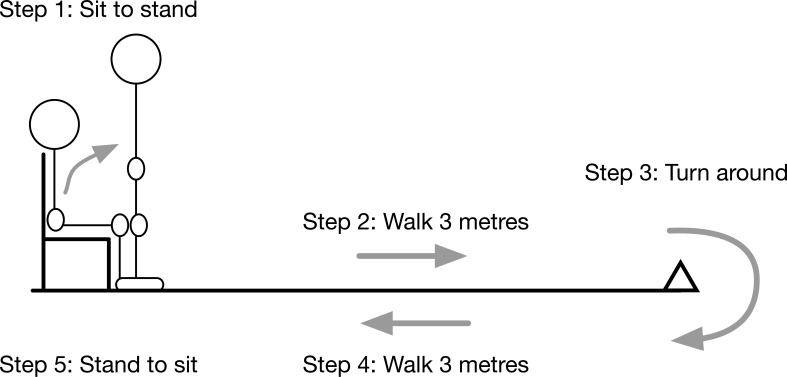
Timed-Up-and-Go test (available on SWASFT electronic Patient Clinical Record).

*Source:* Benavent-Caballer (2016).

The CFS is a simple scale assessing physical frailty. The Timed-Up-and-Go (TUG) (Figure 2) is another, whereby a time of less than 13.5 seconds is associated with a low likelihood of having frailty (Barry, Galvin, Keogh, Horgan, & Fahey, 2014).

There is increasing evidence that recognition of frailty, or pre-frailty, may enable initiation of interventions which may help reverse or postpone the development of severe frailty. In addition, the potential risks of hospital admission for this patient group are well documented (Zisberg, Shadmi, Gur-Yaish, Tonkikh, & Sinoff, 2015).

In 2017–2018, the General Practice Contract for England (NHS England, 2017b) included for the first time a contractual requirement with the aim of improving both the recognition of and response to frailty in primary care. The aim is to proactively identify older people (aged over 64) who are living with severe or moderate frailty (NHS England, 2017b).

Ambulance services are well placed to improve recognition of and response to frailty through recognising patients who are frail, or on the ‘cusp’ of becoming more frail, and accurately communicating this to primary care, whether admitted to hospital or not. Ambulance services can be instrumental in early intervention and decision making. Where important discussions have previously occurred and been documented, for example regarding escalation plans, ambulance clinicians can incorporate these into decision making, and evaluate the pros and cons of hospital admission against other available options. Little has been published regarding delivery of such services.

The widespread delivery of clinical education relating to frailty, provision of easy access to frailty-specific patient assessment tools and establishment of frailty-specific patient care pathways are relatively new developments for UK ambulance services. In the last year, an instructional session covering the concept of frailty, its assessment and the management of patients with frailty has been delivered to SWASFT ambulance clinicians. SWASFT has also introduced an electronic Patient Clinical Record (ePCR) which includes proformas for the completion of the CFS (Figure 1) and TUG (Figure 2) assessment tools, alongside the functionality to email the ePCR directly to GPs and other care providers. It seems intuitive that ambulance clinicians have the potential to identify frailty and its severity, to consider frailty in patient care planning and thereby to influence health outcomes.

## Aim

The aim of the focus groups was to explore and gain insight into the current knowledge, practice and attitudes of ambulance clinicians, with regard to frailty and patients with frailty.

## Methods

An open invitation was sent to SWASFT ‘lead’ clinicians (primarily paramedics) to participate, and all those who volunteered participated. Two focus groups (n = 8; n = 9) were conducted. The groups included registered paramedics (n = 15), some of whom also had additional qualifications/roles, and two emergency care assistants with a particular interest in frailty. Focus group members ranged in experience from those who were instrumental in developing the Trust’s ‘frailty agenda’, to operational clinicians who were the target of these initiatives. There were representatives from across the region served by SWASFT. Most participants had routine responsibility for the assessment and management of patients with frailty. Discussions were semi-structured, and lasted approximately one hour. Participants consented to audio recording of the discussions. These were subsequently analysed by the authors (transcription was sub-contracted to a professional third party). Once the authors were familiar with the transcripts, initial codes were generated, which were then used to identify and name themes. The findings will inform the aims and design of future research.

## Results

Five emergent themes were identified from the analysis (Table 1). These were:

1. individual knowledge and attitudes;
2. factors affecting frailty assessment;
3. information and communication;
4. factors affecting decision making; and
5. systems (care pathways and referrals).

**Table 1.** Themes and sub-themes.

Theme	Sub-theme
*Individual knowledge and attitudes*	Knowledge of frailty condition Function of frailty assessment/interpretation
*Factors affecting frailty assessment*	CFS: ease of use Challenges applying the CFS/education Challenges conducting TUG assessment
*Information and communication*	Contributory information Conveying findings
*Factors affecting decision making*	Human and environmental factors Risks and consequences Clinical role/crossing barriers/joined-up working
*Systems (care pathways and referrals)*	Hospital pathways/integration/medical hierarchy Community pathways/capacity

### Individual knowledge and attitudes

#### Knowledge of frailty condition

Participants noted that although guidelines for the identification and management of frail patients are recent developments within SWASFT, the need to consider factors relating to frailty is not new:

I think we see it; we just haven’t put a title to it. [T1/F2]

Knowledge of frailty varied substantially among participants. However, key features were mentioned:

. . . old or young. And vulnerable to a range of things. [T1/F1]

. . . multiple system failure, where people gradually weaken as they get older. [T1/M1]

. . . the increasing number of co-morbidities, as people generally develop as they get older, has a knock-on effect in frailty. [T1/M2]

It was argued that clinical education can develop a more ‘diverse vocabulary’ [T1/M2] and help to give structure and context to the assessment of frailty. It was felt there should be more training in geriatrics and possibly even ambulance staff who specialise in the care of older adults, ‘with the population getting older’ [T1/M3].

#### Function of frailty assessment/interpretation

Perceptions of why it may be useful to assess frailty in general varied significantly across the groups. One participant noted that formal assessments can contribute to decisions regarding possible community management, arranging supportive care packages and asking for medication reviews. Another noted that there is particular value in using recognised assessment tools, as they can be used for:

validating why I’m trying to negotiate or advocate on their behalf. If that’s required. So that evidence bases my decision . . . for their ongoing care. Which is then shared with the GP and so on and so forth. [T1/M2]

Some spoke of scope to proactively change the way that patients with frailty are managed:

. . . re-selling it as a pre-hab rather than rehab. [T1/F2]

. . . even if we have to go in to ED, it just depends what door we’re going through. At least they know that that patient with maybe severe frailty is arriving in their hospital. If you can expedite their move on to a decent ward . . . then maybe that’ll help. [T1/F1]

It was suggested that a simple, clear score stands out as something to mention to fellow healthcare professionals when patients are conveyed:

. . . when you’re handing over it’s there and you’re going, oh actually. [T2/M2]

. . . it’s transferable then, to somebody else to comprehend later. [T1/M2]

The CFS was seen as suitable to convey succinctly important information about a patient:

It gives you that instant picture of that patient. [T1/F3]

Other potential benefits of common usage were acknowledged:

It’s got much more impact, it’s such a frequent thing. [T1/M2]

I’m also noticing that it is appearing on GP notes more, and I’m now the wiser as to why that’s happening. [T1/F3]

However more than one clinician was sceptical of the value of formal frailty evaluation. One was a strong advocate of the traditional medical model of documentation – ‘put impressions’ [T2/M4] – implying that this approach was in opposition to the use of structured assessment tools.

### Factors affecting frailty assessment

#### Clinical Frailty Scale: ease of use

Some positivity was expressed regarding the CFS and its function. One clinician stated that they use it:

every time I go on the EPCR. [T1/F4]

Such an approach, enabled by the inclusion of the CFS on the ePCR, represents a notable change in traditional assessment and reporting behaviour.

However, the apparently simple nature of the CFS, seen by some as a strength, was perceived as a weakness by others, who disliked its apparent subjectivity:

you could get four or five people doing that score, and each come up with a different number. [T1/M2]

This criticism was associated with a lack of direction regarding information which should be considered when applying the CFS:

there is a vast array of things you can look into to decide if somebody’s frail. [T1/M2]

In addition, there is also the potential to misinterpret evidence, for example to read significance into signs and to overlook potential alternative/extenuating explanations.

#### Challenges applying the Clinical Frailty Scale/education

It was generally recognised that the CFS is designed to appraise a patient’s usual/baseline condition, but that this may be obscured by the reason that the ambulance service has been called:

if we’re dealing with them for an acute reason, we don’t necessarily see their everyday being, if you like. And it might be completely different. [T1/F4]

One clinician reported the effect that falls can have on patients’ confidence and consequently on their presentation to the ambulance crew. Another mentioned the loss of strength that may be experienced after a period lying on the floor, leading to an unrepresentatively high CFS score.

The value of making an ‘educated guess’ [T2/M5] regarding usual levels of functioning was questioned. However, it was also asserted that it is often possible to gather enough information to ‘join up the dots’ [T1/M2]. It was suggested that patients’ care notes (kept in patients’ homes by carers), are inspected to look for patterns to identify the trajectory of their condition. Other useful contextual information was said to include the time of day and whether analgesia had been taken/administered recently (referring to the potential implications of tiredness and pain on mobilisation).

There was recognition that the purpose of the CFS is to broadly categorise:

for us to pick up that they fit into a *big* box. [T2/M5]

and provide a:

holistic view of the patient. [T1/M1]

The point was also made that the CFS was not intended to be used as a stand-alone triage tool to determine what action should be taken for patients, but rather as a guide or ‘case-finding tool’ [T1/M3] to identify patients who may benefit from more detailed assessment.

#### Challenges conducting Timed-Up-and-Go assessment

It was asserted that mobility is often a key consideration when deciding whether a patient can be managed safely at home, but the broad consensus among the groups was that the TUG tool is not widely completed. Reasons given included a lack of understanding of its purpose (in the context of it being one of many assessment tools introduced on the ePCR) and a perception that it is difficult/risky to carry out:

Would you stand not using your hands? [T2/M4]

One clinician referred specifically to the challenges presented by patients who had had a ‘non-injury fall’, saying that:

you get them [to sit] up . . . and then ten minutes later you’re saying, right, get up. [T2/M5]

As with the CFS it was suggested that such difficulties may lead to the temptation to make a guess at it. [T2/M4]

### Information and communication

#### Contributory information

One clinician described a gradual process of discovery, as more time is spent with a patient. Patient care notes were considered valuable. However, frustration was expressed regarding the perceived inability of other care providers to share patient information. Knowledge was limited regarding new developments in this area, such as access to enhanced summary care records from GPs. These have to be accessed proactively. Several contributors recognised that ambulance services do have access to other sorts of information which other care providers do not, for example the possibility of reviewing previous ePCRs:

we’ve been out fifteen times to [xxx] in the last month. [T1/F3]

However, one bemoaned the ability of the ambulance service to ‘accumulate intelligence’ [T1/M2]. Another gave an example of a potential internal information source collated by a clinician who identifies and works with frequent callers. It was suggested that the resulting notes could be made available to operational clinicians and that such additional information could be flagged on the ePCR.

There was discussion regarding whether more point-of-care testing could facilitate the management of patients in their homes, while one contributor responded that such an approach would be problematic and speculated that further testing could be expensive and time consuming and may be inconclusive:

you could still spend an hour or two sorting things out and still end up admitting. [T2/M4]

#### Conveying findings

Some contributors felt that there should be a specific field on the ePCR to document supportive information (including photographs of living conditions): ‘It’s facts, really, isn’t it?’ [T2/M4] or that other, ‘more detailed, perhaps more accurate’ [T1/M1] assessment tools should be made available. One clinician suggested that a short tick box appraisal could convey more objective information – such as PRISMA 7 (Table 2). Another clinician argued that there was scope for the ePCR to better support geriatric assessment in general. This was consistent with other suggestions, that collated information could be presented as a patient profile, or ‘frailty referral’ [T2/M5&F1].

**Table 2.** PRISMA 7 questions.

1	Are you more than 85 years?
2	Male?
3	In general do you have any health problems that require you to limit your activities?
4	Do you need someone to help you on a regular basis?
5	In general do you have any health problems that require you to stay at home?
6	In case of need can you count on someone close to you?
7	Do you regularly use a stick, walker or wheelchair to get about?

*Source:* Raîche, Hébert, & Dubois (2008).

### Factors affecting decision making

#### Human and environmental factors

Motivation to pursue patient referrals was said to be affected by various ‘human factors’. Geography was said to influence behaviour:

big city mentality is, you know, I’m three minutes down the road, stick them in the back, let’s take them in. Done. [T2/M6]

whereas:

if you’re more rural . . . you’ve got an hour, say, to get to your receiving A&E department. You’re gonna give a little bit more time, thought, in well, actually can I save myself an hour and half, two hours, by staying here for twenty minutes. [T2/M6]

One clinician added that:

the pressure being brought to bear at this particular moment on conveyance times, turnaround cycle times . . . [T2/M6]

. . . impacts on the uptake of services which are perceived to take time, that clinicians will follow: ‘paths of least resistance’ [T2/M5] and that:

ten, eleven hour shifts with minimum breaks . . . can impact on the likelihood of trying to arrange options which require additional thought. [T2/M5]

It was observed that some previously established care pathways are no longer available: ‘we’ve had that all blocked’ [T1/F2]. It was suggested that easy access to information about which care pathways are available locally would help: ‘It optimises our attendance, optimises your time’ [F1/M2] and would end reliance on local knowledge. The facility to search for appropriate pathways in specific locations is available on the ePCR, but there appeared to be little awareness of/faith in this system:

Is that just through emails you’d get that sort of information? Or is there something, a central way . . .? [T2/F1]

However, past experience was also referred to:

if you have successful uses of it, you use it more. [T2/M5]

Another complicating factor was said to be stigma attached to ‘phoning certain units and asking for help’ [T2/M1], or contacting certain ambulance staff with an advisory role.

#### Risks and consequences

One clinician was concerned by the perceived risks involved in managing patients at home; risks to patients (that they may deteriorate in a relatively unmonitored environment) and risks to clinicians (that they may be perceived to have failed to anticipate such deterioration and are therefore held responsible):

you’re not going to get into trouble for taking someone in. [T2/M5]

Another took the view that a certain amount of risk is acceptable, if trying to act in patients’ best interests: ‘sometimes things go wrong’ [T2/M1]. He suggested that such risk is acceptable/reduced if it is believed that a patient is unlikely to receive additional treatment in hospital, beyond that which they could receive in the community. The perspective that ‘more shared ownership of the decision’ [T2/F1] could mitigate risk, however, was also put forward.

One contributor suggested that knowledge of the risks to frail patients of being admitted to hospital is becoming more widespread:

admitting a patient into hospital can be to their detriment . . . [T1/M3]

. . . and was said to counter a more traditional perspective that it is always best to:

want these old people in hospital because I want them, you know, to be safe. [T1/F3]

This was supported by recognition of evidence that people with dementia, who are particularly prone to frailty, are:

better not to move from their environment. [T1/F4]

One participant described trying to change this traditional culture of hospitalisation, during admission:

We’re trying to stop them bringing in their night clothes; stop them from leaving everything at home; no we mustn’t take our wheel chairs or . . . Zimmer frames, or . . . medication cos it’ll get lost. We want all that to go in, and wear your day clothes and be prepared to leave and come back home tomorrow. But that’s trying to change a whole generation’s mind-set that they’re going in for 10 days. [T1/M2]

Regarding discharge from hospital, it was implied that family members’ expectations need to be managed, as patients may be perceived to have been discharged ‘too early’ if they are unaware of the potential benefits of timely transfer to the home environment. However, one group member was distressed that:

the number of . . . failed discharges that you go to is just phenomenal. [T1/M1]

It was observed that, although discharge from hospital may often appear to be best for patients, such rationale may rely on the provision of sufficient support once they arrive home, and it was asserted that ‘reablement’ teams are often overstretched and/or not available during nights and weekends:

they’re so busy they’re finding by Wednesday they can’t take any more for the rest of the week. [T1/M3]

It was perceived that, as a consequence, patients are sometimes managed at home in suboptimal conditions, affecting their recovery and resulting in preventable re-admissions to hospital. Further complications of the discharge process were attributed to medication changes made in hospital (including enforcing regimen that weren’t being adhered to at home), the impact of which only becomes evident when the patient is discharged.

#### Clinical role/crossing barriers/joined-up working

Many of the discussions about the management of frail patients illustrated debates which are taking place more widely in the ambulance service. These include efforts and provision to facilitate the appropriate treatment of patients in the community. One clinician made the point that if we are ‘crossing that barrier’ [T1/M2] (away from the ‘traditional’ ambulance role) we may in fact be duplicating effort:

all these people and these disciplines are walking up the same path to see the same people. [T1/M2]

Another contributor offered a solution to this perceived problem, which they described as ‘joined-up working’ [T1/F3]. There was qualified support for expanding the role of the ambulance service:

I’m not trying to dilute what SWASFT does as a core business, but we’re overlaying so many of these boundaries, we’ve gotta have a workforce that crosses that as well. And is capable of speaking the same language. [T1/M2]

Another added:

If you could just have a bit more multidisciplinary working, with a single point of access, and we could do the triaging then. That’s why I think walk-in centres are a way forward . . . we will then send you to the most appropriate clinician. [T2/M4]

However, the same person also cautioned:

The ambulance service has to be careful it doesn’t re-create the NHS in microcosm . . . we’re just trying to deal with groups of patients in-house . . . that there’s already a mechanism for really. [T2/M4]

Some paramedics have undertaken additional training, primarily to support the wider ambulance service in delivering appropriate urgent care for patients out of hospital. One contributor argued that these Specialist Paramedics in Urgent and Emergency Care (SPUECs) are ideally placed to ‘talk to, which helps your go/no go decisions’ [T2/M4] and that they can offer some flexibility and be booked to attend patients at a later time, while another noted SPUECs’ enhanced ability to treat some infections and wounds at home. However, concerns were expressed about the availability of SPUECs and the future of the role. This was discussed in the context of uncertainty over the strategic direction of the ambulance service.

Perhaps the least traditional activity mentioned, with regard to ambulance work, was a pilot scheme to pre-emptively visit and support vulnerable patients in care homes late in each week. It was said to have successfully reduced calls made to the ambulance service during the weekend. This proactive approach was viewed positively:

rather than continually reflecting back and looking at how we should have done. [T1/M2]

It was reported that in one area SPUECs have been involved in a trial to provide items such as commodes and walking frames to patients. This was designed to meet immediate needs which may enable patients to be managed at home at times when occupational therapy referrals are not available. One SPUEC also said:

I can do far more when I’m working in primary care . . . [T2/M1]

. . . although he added that such activities take additional time.

### Systems (care pathways and referrals)

#### Hospital pathways/integration/medical hierarchy

One clinician supported the idea that ‘discharge planning starts with you’ [T1/M1], expressing the value of conveying an impression of a patient’s home circumstances when taking them to hospital. However, even where arrangements are in place to enable direct referral to assessment wards, they were said to be inconsistently available. Some were said to only be open for SPUECs to use. Elsewhere, the determining factor was reported to be:

the person you happen to speak to at the end of the phone. [T1/M1]

Various local arrangements to improve hospital admission procedures were discussed, including an alert system for specialists to come to ED to ‘clerk’ patients. Another was a ‘Community Assessment Panel’ [T2/M1], designed for the:

same-day turnaround of people who . . . they can shift. [T2/M1]

One SPUEC said that he finds that when working for the ambulance service he would usually ‘have to go through ED’ [T1/M2], whereas when he is employed in a primary care role:

they go straight to the clinician that they need to go to. [T1/M2]

One person also reported a qualification to the idea that avoiding the ED is always best for frail patients. They had been advised by an acute GP that if a patient is admitted to a medical ward, they are more likely to ‘get into the process’ [T2/M4], implying that under some circumstances, ED may be the more efficient option.

#### Community pathways/capacity

Examples were given of telephone call-centres which can facilitate referrals to various different community services such as occupational therapy, medication reviews and falls and rapid response teams: ‘one-stop-shop units’ [T1/F1]. However, one clinician expressed his belief that provision of these services, usually known as ‘Single Points of Access’ (SPoA), is inconsistent across the region. Another believed that plans are underway in one area to enable referrals for Comprehensive Geriatric Assessment (CGA) within a day or two. A general sense was conveyed that more referral pathways are being made available to ambulance clinicians, some of which are ‘specifically for frail old people’ [T1/F1], but that there are inadequate care pathways available in some areas and inconsistent access to those that do exist. One SPUEC described having to exercise:

my sort of butt-headedness . . . [to] sort out the solutions. [T1/M2]

Services such as ‘Rapid Response’ teams, ‘Acute Care at Home’ and ‘Early Intervention Service’ were mentioned as short notice providers of short-term care which can enable a patient to stay in their home, rather than being taken to hospital (to enable their health to improve and/or a longer-term care package to be put in place). Such services were said to include taking ‘bloods’ and the administration of intravenous antibiotics – potentially blurring the line between hospital and home. Once again, however, availability was said to be limited:

they’re under-staffed and there aren’t enough of them so you end up, cos there’s nobody there that can come and deal with it, you end up taking Mrs [xxx] in because there’s nowhere else to take her. [T1/F4]

One clinician spoke of the services available through Primary Care:

If I . . . leave a patient at home I’ll always email my Acute Referral Form off to the GP practice . . . I’ve referred patients to GPs, either for a GP follow-up or possibly physios or OTs to visit. [T1/M2]

Another said:

I think there’s a bit more knowledge, now, about us being able to refer specifically for a medication review or for a comprehensive geriatric assessment . . . [T1/F1]

. . . while recognising that to make such referrals staff might need to know

where to ask for that and what it does involve and what the patient may get out of it. [T1/F1]

Minor Injury Units were also mentioned as a resource which may support the management of patients in the community, although their limited remit (injury, rather than illness) was seen to restrict their usefulness. Difficulties in finding appropriate available care pathways for patients led one clinician to conclude:

it seems to me that we’re the only people trying to keep people *out* of ED. [T2/M4]

Another summarised her perspective regarding support to manage frail patients at home:

it’s a really good idea and it’s a good initiative, but it’s not being backed up well enough behind . . . [T1/F4]

. . . apparently summing up perceived limitations regarding frailty care pathways.

## Discussion

Recognition and prioritisation of frailty as a condition has gained widespread recognition across healthcare systems in recent years. The professional training received by most ambulance personnel is therefore unlikely to have conveyed the evolving models used to describe frailty, the usefulness and practicality of different approaches to its assessment or the complexity and subtleties of decision making now understood to offer optimal care for those with frailty. These focus groups have provided evidence of the knowledge and attitudes which clinicians in SWASFT have acquired and the practice which they have developed to-date, with regard to frailty.

Among ambulance clinicians who have responsibility for identifying and managing patients with frailty, there appears to be only modest understanding of the medicalised theories of frailty. Direct knowledge of common concepts of frailty such as decreased physiological reserves in multiple systems, increased vulnerability to stressors and limited capacity for maintaining homeostasis (Clegg et al., 2013) appeared to be limited to one or two participants, although insight was often implied by others. In the opinion of the authors, taken as a whole, a good breadth of knowledge regarding the appropriate assessment and management of patients with frailty was demonstrated. However, familiarity with current guidance seemed to vary substantially between individuals, apparently hampered by constantly evolving services and geographical variation.

The introduction of the CFS on SWASFT’s ePCR has led to a significant development in patient assessment. A broad measure of the level of frailty exhibited by patients is being routinely reported using a recognised frailty screening tool. The perceived value of this practice was generally positive, but did vary. Concerns related to a lack of guidance regarding application, purpose, the requirement to appraise usual functioning (even if masked by acute presentation) and apparent subjectivity/over-simplicity. Positive feedback included instant impact, transferability of meaning and the provision of evidence.

Doubts were expressed regarding the suitability of the TUG assessment tool for out-of-hospital urgent care use (a perspective reflected by a much lower completion rate compared to CFS). It was clear, however, that in the absence of a practical mobility assessment tool, informal impressions of mobility are nevertheless routinely recorded. It is possible that the clear variability of underpinning knowledge regarding frailty and its assessment may correspond with a lack of consistency in the application of assessment tools, which may limit the reliability of their results.

The range of information which can contribute to evaluation of frailty was acknowledged and appeals were made for the ePCR to be given the functionality to prompt the systematic recording of greater detail, possibly including further assessment tools. There was also broad agreement that better information sharing within SWASFT and from external organisations such as primary care providers would support patient assessment and management. A similar rationale underpins recent changes to the GP contract.

Many of the care pathways discussed seem likely to support the goals of the wider health service for patients with frailty, including the new GP contract. The effective implementation of this plan would see all patients with moderate or severe frailty identified and provided with the opportunity to receive evidence-based interventions, known to reduce their risks due to frailty and potentially help reverse frailty. Also, improved information sharing would inform healthcare professionals of frailty status, so that it may be incorporated into risk/benefit assessments.

Ambulance services may also support the identification of patients who may require CGA – the ‘gold standard’ for the care of elderly patients with frailty. CGA is a multidisciplinary process which can be initiated by various healthcare providers (primary/community/acute). It aims to identify the particular combination of deficits (leading to frailty) and assets (contributing to resilience) that a person has (Åhlund, Bäck, Öberg, & Ekerstad, 2017). The most effective interventions arising from CGAs seem to be multi-domain in nature (Dedeyne, Deschodt, Verschueren, Tournoy, & Gielen, 2017). The effectiveness of these interventions may differ depending on the degree of frailty (Theou et al., 2017). Physical exercise, for example resistance training, seems to be particularly valuable (Puts et al., 2017).

Discussions regarding the management of patients with frailty reflected wider debates within the ambulance service concerning the provision of appropriate care for those whose presentations are of an urgent, rather than time-critical nature. There was some disagreement about the lengths which should be gone to facilitate the safe care of patients in their homes. Conversation regarding SPUECs illustrated ways in which the ambulance service can directly meet patients’ needs. Referrals to other frailty orientated services such as primary care/community services, when hospital admission would otherwise be likely, or to elderly assessment units/specialists when conveyance to hospital is the appropriate intervention were also discussed. While there was support for such activity and for the ability of ambulance staff to contribute in a way that would meet perceived need, there was also acknowledgement that care must be taken to avoid duplication of effort and to minimise the inefficient ‘tying-up’ of ambulance resources.

A sense was conveyed that the ambulance service can (and to a degree already does) provide an effective link between patients and a range of different care providers (Goldstein, McVey, & Ackroyd-Stolarz, 2016). It seems clear that the facility in SWASFT to email patient report forms to GPs is already well used, despite being a new development, and that it is being used to request specific services. However, the impression was also conveyed that barriers to accessing potential alternative care pathways were the rule rather than the exception. Examples given included communication difficulties, the narrow remit of Minor Injury Units and the inability to refer patients directly to community hospitals or nursing homes.

A number of initiatives were mentioned regarding how ambulance services can contribute to efficient and appropriate hospital care for patients with frailty. There was recognition of the often unique access that ambulance clinicians have to first-hand knowledge of patients’ living conditions and the importance of conveying such impressions to hospital staff, who will often read ambulance records as patients move through the hospital system. There was also an evident awareness of the impact that the approach to ‘packing’ for hospital can have on patient outcomes – particularly in challenging ‘pyjama paralysis’, by encouraging patients to take day clothes and mobility aids with them.

There is some evidence that various, generally small-scale schemes, involving ambulance clinicians elsewhere in England, have been introduced specifically to address the needs of patients with frailty (British Geriatric Society, 2016), although no evaluation of these has been published. In Canada, efforts to introduce the systematic assessment of older patients by paramedics have been evaluated, but appear to be time intensive (Goldstein et al., 2014), and in Australia a recent report described support among paramedic students for the use of two frailty screening tools, each of which took about 13 minutes to complete (Harris, Lucas, Eyles, & Parker, 2018).

Different geographical regions have adapted differently to changing needs, resulting in considerable diversity of services. A range of local formal and informal arrangements for admitting patients directly to medical, elderly and even frailty assessment units clearly exists. However, such schemes vary across the south-west region and appear to be widely affected by variability of access. Recent ambulance service initiatives to provide up-to-date information about what services are available, where and when, for example with an online directory of services, do not yet appear to be reaching their full potential regarding staff awareness and utilisation.

Elderly patients with frailty may be in contact with many health and social care professionals and are vulnerable to risks associated with transitions between care settings (Baillie et al., 2014). Perceived restrictions to the availability of and access to community and acute care pathways for ambulance personnel are evidence of insufficient capacity and trust at the interface between vertically integrated elements of the care system. In addition, these barriers may restrict healthcare options available to patients and thereby the ability of patients, their families and their carers to influence care choices (D’Avanzo et al., 2017).

### Strengths and limitations

This qualitative service evaluation explores perceptions and attitudes towards subject matter which has received little attention of this kind previously. It does however build upon a previous study of the experiences of patients with frailty attended by ambulance personnel (Robertson & Cooke, 2016).

A significant number of views are represented, from across the region, and from personnel with a broad range of clinical experience; although senior clinicians made a disproportionately large contribution to the recorded discourse. Groups were self-selecting, favouring those with an established interest. Sessions were held after a Continuing Professional Development event on the subject of frailty. However, discussions were free, wide-ranging and open.

## Conclusions

This service evaluation provides insight regarding the Ambulance Trust in which it was conducted. Significant ambulance clinician knowledge of service initiatives to develop the understanding, assessment and management of patients with frailty was evident within the two focus groups. However, levels of familiarity with these initiatives appeared to vary significantly within the groups. A diversity of opinion was offered regarding the suitability of the assessment tools provided on the ePCR, although a shift towards the routine recording of such measurements is evidently underway. Debate surrounding the lengths to which ambulance personnel should go to facilitate the management of patients in their own homes, or to expedite a smooth transit to and through frailty orientated hospital care, reflected ongoing discussions regarding the strategic direction of ambulance service activity. However, such care pathways appear to be restricted by inconsistent knowledge of them and/or limitations in their availability. Variation in knowledge of frailty, its assessment and management, reflected the emerging nature of the condition both academically and clinically.

## Author contributions

All authors drafted/revised this manuscript and approved the final version.

## Conflict of interest

None declared.

## Ethics

Not required.

## Funding

This service evaluation has been supported by NIHR Research Capacity Funding, with the aim of informing future research planning.
